# Spatially Resolved Transcriptomic Signatures of Hippocampal Subregions and *Arc*-Expressing Ensembles in Active Place Avoidance Memory

**DOI:** 10.1101/2023.12.30.573225

**Published:** 2024-01-01

**Authors:** Isaac Vingan, Victoria Sook Keng Tung, Shwetha Phatarpekar, A. Iván Hernández, Oleg V. Evgrafov, Juan Marcos Alarcon

**Affiliations:** 1 School of Graduates Studies, Program in Neural and Behavioral Sciences, State University of New York, Downstate Health Sciences University, Brooklyn, NY, USA; 2 School of Graduates Studies, Program in Molecular and Cell Biology, State University of New York, Downstate Health Sciences University, Brooklyn, NY, USA; 3 Institute of Genomic Health, State University of New York, Downstate Health Sciences University, Brooklyn, NY, USA; 4 Department of Pathology, State University of New York, Downstate Health Sciences University, Brooklyn, NY, USA; 5 Department of Cell Biology, State University of New York, Downstate Health Sciences University, Brooklyn, NY, USA; 6 The Robert F. Furchgott Center for Neural & Behavioral Science, State University of New York, Downstate Health Sciences University, Brooklyn, NY, USA

**Keywords:** Spatial Transcriptomics, Gene Expression, Memory, Hippocampus, CA1, CA3, Dentate Gyrus, Immediate Early Gene, IEG-Expressing Ensemble, Memory-associated Neuronal Ensemble

## Abstract

The rodent hippocampus is a spatially organized neuronal network that supports the formation of spatial and episodic memories. We conducted bulk RNA sequencing and spatial transcriptomics experiments to measure gene expression changes in the dorsal hippocampus following recall of active place avoidance memory. Our analysis focused on two specific levels of spatial resolution: hippocampal subregions and Immediate Early Gene (IEG) expressing cellular assemblies. Through bulk RNA sequencing, we examined the gene expression changes following memory recall across the functionally distinct subregions of the dorsal hippocampus. We found that training induced differentially expressed genes (DEGs) in the CA1 and CA3 hippocampal subregions were enriched with genes involved in synaptic transmission and synaptic plasticity, while DEGs in the dentate gyrus (DG) were enriched with genes involved in energy balance and ribosomal function. Through spatial transcriptomics, we examined gene expression changes following memory recall in putative memory-associated neuronal ensembles marked by the expression of the IEGs *Arc*, *Egr1*, and *c-Jun*. Within samples from both trained and untrained mice, the subpopulations of spatial transcriptomic spots marked by these IEGs were functionally and spatially distinct from one another. In only the hippocampus of trained mice, DEGs detected between Arc+ and Arc− spots were enriched in several memory-related gene ontology terms, including “regulation of synaptic plasticity” and “memory.” Our results suggest that memory recall is supported by region-specific gene expression changes and transcriptionally distinct IEG expressing ensembles of neurons in the hippocampus.

## INTRODUCTION

The neural operations supporting memory require the involvement of various brain systems interconnected through neural networks [1]. The activity of these networks is fine-tuned by experience through the selective recruitment of ensembles of neurons ascribed to contain the bits of information associated with memory [2]. Neuronal ensemble activity is shaped by synaptic plasticity mechanisms that modulate the weight and efficacy of the ensemble’s synaptic connections [3, 4]. Changes in gene expression are a key underlying mechanism in synaptic plasticity [5], and recent studies have identified multiple profiles of gene expression associated with memory [6–8].

In the rodent hippocampus, encoding of spatial memory information is supported by the diversity of synaptic computations within and across its sub-fields [9, 10]. Each hippocampal subregion (i.e. Dentate Gyrus, CA1, CA2, CA3) shows distinct patterns of activity to process spatial information and to form or retrieve memories [11–16]. At the regional level, patterns of activity are conferred through the combination of cyto-architecture, local synaptic circuitry, neuronal cell types and synaptic inputs unique to each region [7, 13, 17–26]. At the single-cell level, the functional state of hippocampal neurons is defined by connectivity, hippocampal subregional location, and experience-dependent recruitment into a memory ensemble [2, 27].

The investigation of memory across molecular and functional levels necessitates an approach that connects the scales of hippocampal organization between cellular mechanisms and networks of cells. While the functional contribution of individual genes that underly synaptic plasticity at the cellular level are well-established [4, 28–31], it remains unclear what changes in gene expression across hippocampal subregions and subpopulations of the memory-associated neuronal ensembles characterize memory in the hippocampus.

Memory-associated neuronal ensembles are a sparsely distributed population of neurons that strongly activate together during the acquisition and recall of a memory, and are thought to encode the particular bits of information relevant to that memory [20, 32, 33]. Numerous studies have identified Immediate Early Genes (IEGs) such as *Arc*, *c-Fos*, *Egr1*, *c-Jun*, *Npas4*, *Bdnf*, and *Fmrp* to characterize memory-associated neuronal ensembles [2, 34–36]. In this study, the location of memory-associated neuronal ensembles is inferred from the expression of IEGs in spatial transcriptomic spots. The expression of *Arc* mRNA has been used to tag and characterize the properties of memory-associated neuronal ensembles [37–39]. This proxy for the memory-associated neuronal ensemble was used to explore the transcriptomic changes supporting memory recall in the hippocampus. We characterized changes in gene expression across the major subregions of the transverse dorsal hippocampus using bulk RNA sequencing of microdissected dorsal hippocampal subregions, and investigated the spatial distribution of IEG-associated gene expression through a spatial transcriptomic study of coronal dorsal hippocampal slices.

## RESULTS

### APA trained mice exhibit place avoidance memory

**Figure 1.**
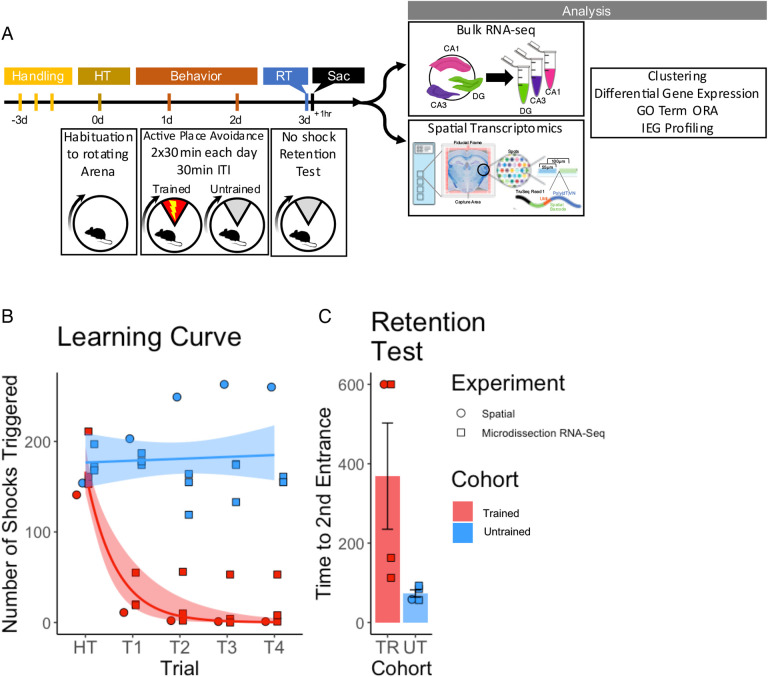
Trained mice learned to avoid the location of an unmarked shock zone A) Experimental timeline. Mice were trained in the active place avoidance paradigm over the course of 4 days following 3 days of handling. On day 0 they were habituated to a rotating arena with no active shock zone (HT). On day 1 and 2 trained mice received two 30-minute trials with an active shock zone. Untrained mice were not exposed to an active shock zone. On day 3 all mice received a 10-minute retention test trial (exposed to the rotating arena with no active shock zone). Mice were sacrificed 60 minutes following the end of the retention test, brains were collected for further processing. B) Training performance measured as number of shocks triggered. Trained mice learned to avoid the location of the shock zone indicated by the decreased number of shocked triggered. Exponential fit learning curves included with shaded error bars (SE). C) Memory performance measured as time to second entrance in the retention test trial. Trained mice showed higher time to second entrance during the retention test. Trained mean 375s ± 70.4 (SE), Untrained mean 58.7s ± 8.51 (SE).

To investigate gene expression changes induced by active place avoidance (APA) training in the hippocampal network, we conducted bulk RNA-sequencing and spatial transcriptomics (see Figure 1A for experimental timeline). In the APA, mice learn to avoid a 60° shock zone on a rotating circular arena [40, 41]. Mice trained in the APA showed a decrease in the number of shocks delivered during the acquisition trials (Figure 1B) and longer re-entry times to the shock zone during the memory retention test trials (Figure 1C).

### Bulk RNA-seq transcriptomic profiling DG, CA3 and CA1 separate trained from untrained cohorts

**Figure 2.**
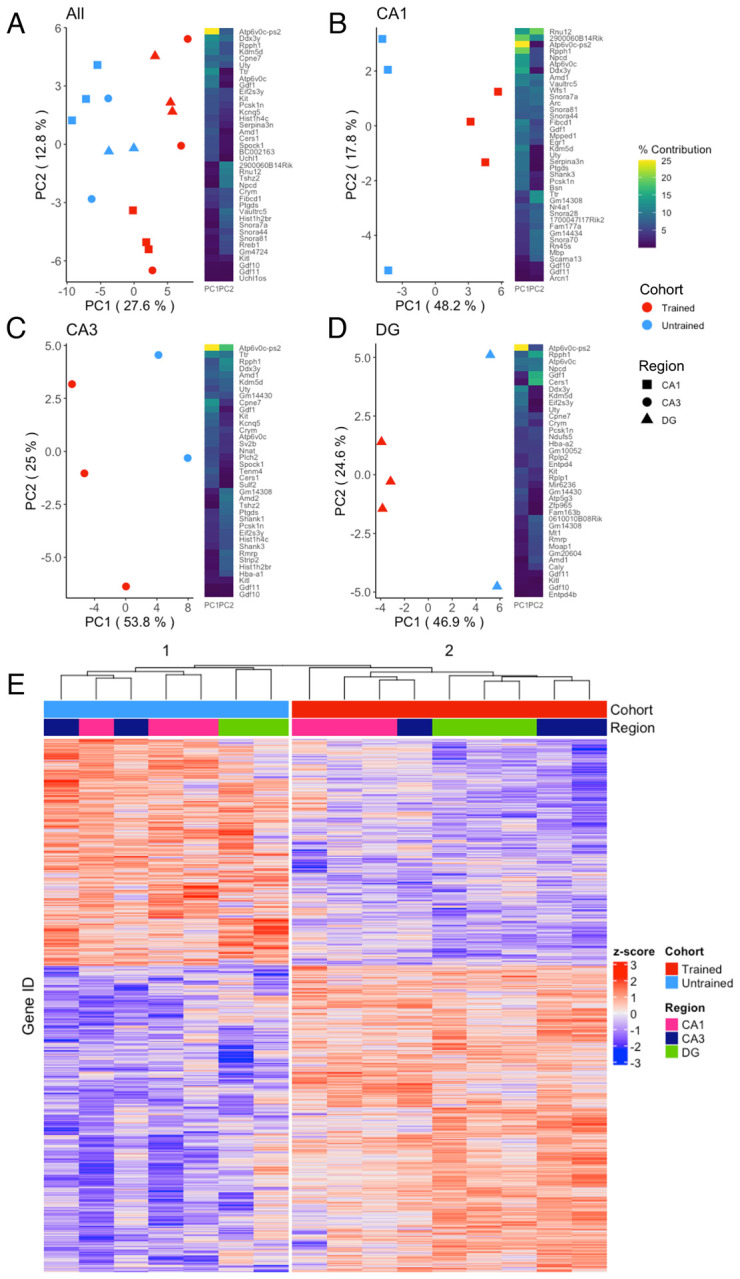
APA training results in substantial changes in hippocampal subregion gene expression profiles A–D) Left, principal component analyses of all hippocampal subregions (A), CA1 (B), CA3 (C), DG (D) illustrate a clear separation between trained and untrained samples along the axis that explains the most variance. Right, heatmap with top 20 contributing genes to the first two principal components of each are reported in the heat map to the right of each plot. Sample size for the CA1 was 3 trained and 3 untrained (B). Sample size for the CA3 was 3 trained and 2 untrained (C). And Sample size for the DG was 3 trained and 2 untrained (D). E) Normalized gene expression of the top 2000 most significant DEGs. Hierarchical clustering of gene expression profiles groups samples by behavioral cohort.

To explore the molecular mechanisms underlying memory recall across the dorsal hippocampus, bulk RNA-sequencing was performed on microdissected DG, CA3 and CA1 hippocampal subregions. Principal component analysis (PCA) from all 3 subregions combined reveals a separation of samples by behavioral cohort (Figure 2A), indicating that differences in training contributes substantially to the overall variability of gene expression in our samples. Separation between samples is further accentuated when PCA is performed separately on data from each subregion (Figure 2C–D). Consistently, hierarchical clustering of gene expression profiles reliably grouped samples by their behavioral cohort (Figure 2E).

### Synaptic plasticity and learning and memory-related biological processes are regionally enriched

**Figure 3.**
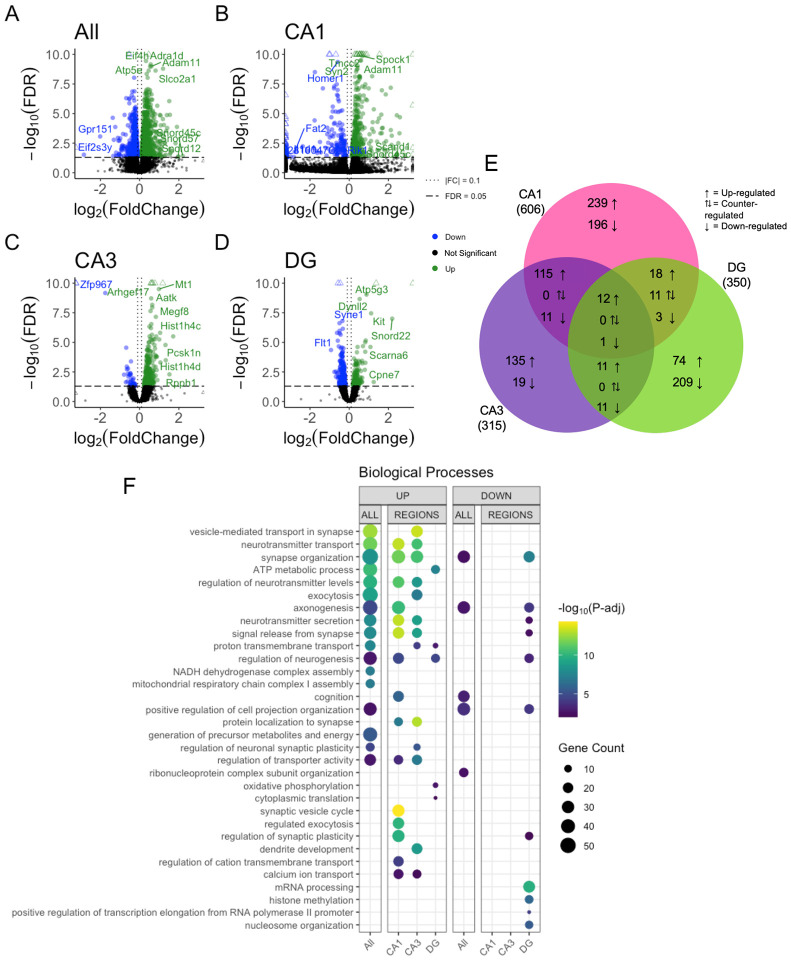
Bulk RNA-seq of hippocampal subregions reveals similar expression profiles in CA1 and CA3 regions in trained samples A–D) Volcano plot of DEGs between trained and untrained in the CA1, CA3 and DG samples, combined and individually. See Supplemental Figure 1A–D for full sized plots. E) Overlaps in regional DEGs demonstrate greatest similarity between CA1 and CA3 subregions. DEGs from each regional analysis were stratified by direction of fold change. Overlaps in DEGs between the CA3 and CA1 subregions were significant (Fisher’s Exact Test). See Supplemental Figure 1E for further details. F) Biological processes overrepresented amongst DEGs detected between samples from trained and untrained animals for CA1, CA3 and DG subregions (all combined and separated). Dot color reflects the statistical significance (−log_10_(FDR)) of the biological process enrichment. Dot size reflects the number of detected DEGs mapped to the genes involved in a given biological process. See Supplemental Figure 1 for enrichment of Cellular Component and Molecular Function GO terms.

We detected 1663, 606, 315 and 350 differentially expressed genes (DEGs) (adjusted p-value < 0.05) between trained and untrained samples for the combined hippocampal subregions and separated CA1, CA3, and DG subregions, respectively (Figure 3A–D). 966 genes were upregulated and 697 were downregulated in all hippocampal subregions. In CA1 393 genes were upregulated and 213 were downregulated, in CA3 273 genes were upregulated and 42 were downregulated, and in DG 117 genes were upregulated and 223 were downregulated. In each comparison the majority of highly significant DEGs (FDR < 0.005) exhibited a modest effect size (|log_2_ fold| < 1.0). Among these DEGs in the combined subregions with a high significance and modest effect size, there are the genes *Ywhag*, *Bsn*, and *Nup153*, which are related to cell signaling and morphogenesis [42–44]. In the CA1, we found *Camk2n1*, *Shank3*, *Shank1*, and *Snap* 25 which are related to synaptic signaling and plasticity [45–48]. We found *Prckg*, Ttr, and *Snhg11* in the CA3 which are related to memory [49–51]. *Kit*, *Srrm1*, and *Syn2*, found in the DG, are related to cell morphogenesis and synaptic signaling [52–54].

To further elucidate the distribution of DEGs across hippocampal subregions we investigated the overlap of significant DEGs (FDR < 0.05). We found the largest overlap of upregulated DEGs between the CA3 and CA1 subregions with a total of 127 intersecting genes (Figure 3E). The genes *Snap25, Shank1, Shank 3, Bsn and Ywhag* were identified in the overlap between the upregulated DEGs of CA3 and CA1 and are known for their involvement in synaptic plasticity. *Ywhag* was also found to be upregulated in the overlap of all three subregions.

Next, we performed Gene Ontology (GO) enrichment analyses to annotate each subregion (i.e., DG, CA3 and CA1) with GO terms overrepresented amongst the DEGs detected in each subregional analysis [55]. In all hippocampal regions combined, we found DEGs were significantly enriched with genes involved in synaptic machinery (Figure 3F & Supplemental Figure 1F,G). Upregulated DEGs detected in both the CA3 and CA1 were enriched with genes involved in synaptic transmission. Upregulated DEGs detected in the DG were enriched with genes involved in neurogenesis and ATP production. In the DG downregulated DEGs were involved in transcriptional machinery and epigenetic modifications (Figure 3F). Downregulated DEGs in the CA1 and CA3 were not significantly enriched with any biological processes. These data reveal a spatial distribution of gene expression across the dorsal hippocampus, defined by a greater degree of similarity between the CA1 and CA3 than between either of these subregions and the DG.

### Differences in the spatial distribution of hippocampal gene expression between trained and untrained animals

**Figure 4.**
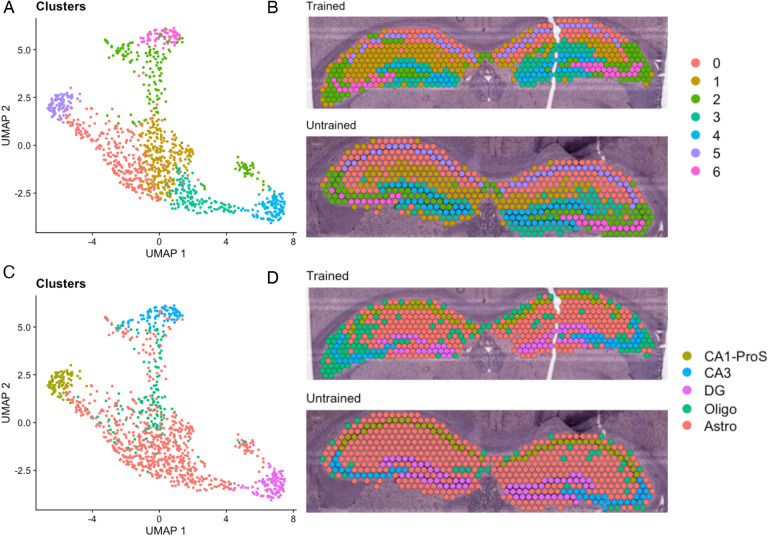
Clustering and cell-type annotation reflect anatomical boundaries of the hippocampal subregions A,B) UMAP plot (left) and spatial transcriptomic maps of hippocampal spots (right) from trained and untrained mouse samples. Color reflects clustering of hippocampal spots. C,D) UMAP plot (left) and cell-type annotated spatial transcriptomic maps of hippocampal spots (right) in samples from the trained and untrained mouse.

To further investigate the spatial distribution of DEGs associated with APA memory across the hippocampus, we performed spatial transcriptomics on coronal sections containing the dorsal hippocampus from one trained and one untrained mouse. Computational analyses of integrated capture spots in the hippocampus of trained and untrained samples reveals distinct clusters (seen in a Uniform Manifold Approximation and Projection (UMAP) plot) which map along anatomical boundaries in a spatial transcriptomic plot (Figure 4A,B). Spatial transcriptomic data were integrated with the Allen Brain Atlas to computationally annotate each spot with the cell-type most prominently detected [56] (Figure 4C,D).

**Figure 5.**
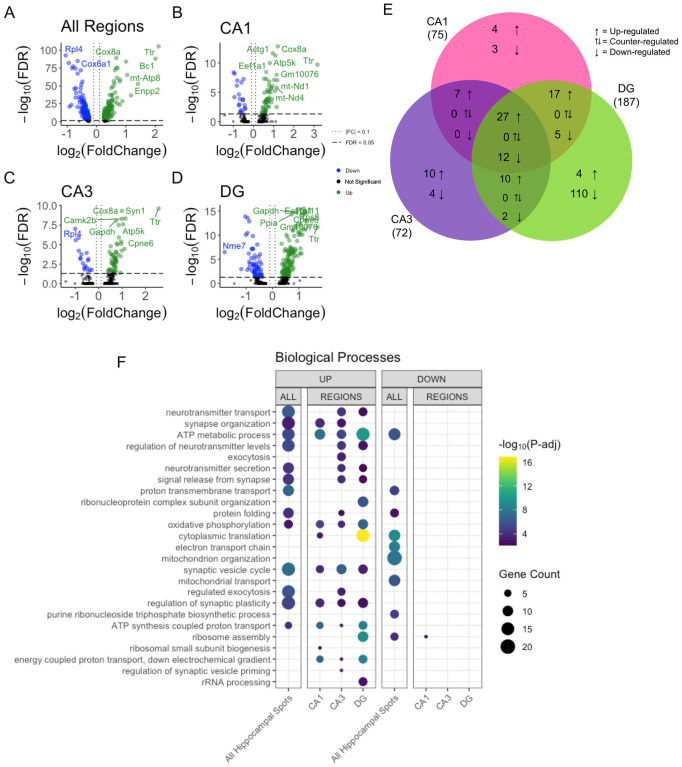
DEGs detected between trained and untrained animals within hippocampal subregions using spatial transcriptomics are enriched with genes involved in similar biological processes to those seen in bulk RNA-seq A–D) Volcano plot of DEGs between all hippocampal (A), CA1 (B), CA3 (C), and DG (D) cell-layer spots from trained and untrained mice. E) Overlap of regionally detected DEGs stratified by direction of fold change. Overlaps were tested with Fisher’s exact test. See Supplemental Figure 2A for further details. F) Biological processes overrepresented amongst DEGs detected between samples from trained and untrained animals for hippocampal samples or all hippocampal spots, and spots from the separated CA1, CA3 and DG cell layers. Dot color reflects the statistical significance (−log_10_(FDR)) of the biological process enrichment. Dot size reflects the number of detected DEGs mapped to the genes involved in a given biological process. See Supplemental Figure 2 for enrichment of Cellular Component and Molecular Function GO terms.

We detected 352 DEGs in all hippocampal spots (205 upregulated and 147 downregulated genes) between trained and untrained samples. 75 DEGs in the CA1 cell layer (55 upregulated and 20 downregulated genes), 72 DEGs in the CA3 cell layer (54 upregulated and 18 downregulated genes), and 187 DEGs in the DG cell layer (129 upregulated and 58 downregulated genes) (Figure 5 A–D). Genes *Cnih2, Syn1*, and *Camk2b*, were differentially expressed in the CA1, CA3, and DG, respectively. These genes are involved in synaptic plasticity and learning and memory [57–59].

In the comparison of DEGs between all hippocampal spots, GO enrichment analyses identified biological processes related to synaptic plasticity and synaptic signaling (Figure 5F & Supplemental Figure 2B,C). In the CA1 and CA3 subregions, we found upregulation of genes involved in biological processes also related to synaptic plasticity and synaptic signaling (Figure 5F). In the DG upregulated DEGs were enriched in biological processes involved in protein expression and ribosomal function (Figure 5F). CA1 was the only subregion where downregulated DEGs show enrichment for biological processes. Our results show a high similarity in the biological functions overrepresented in both the spatial transcriptomic and bulk RNA sequencing differential gene expression analyses between trained and untrained samples.

### Arc-expressing hippocampal spots exhibit changes in the expression of memory-associated genes and biological processes

**Figure 6.**
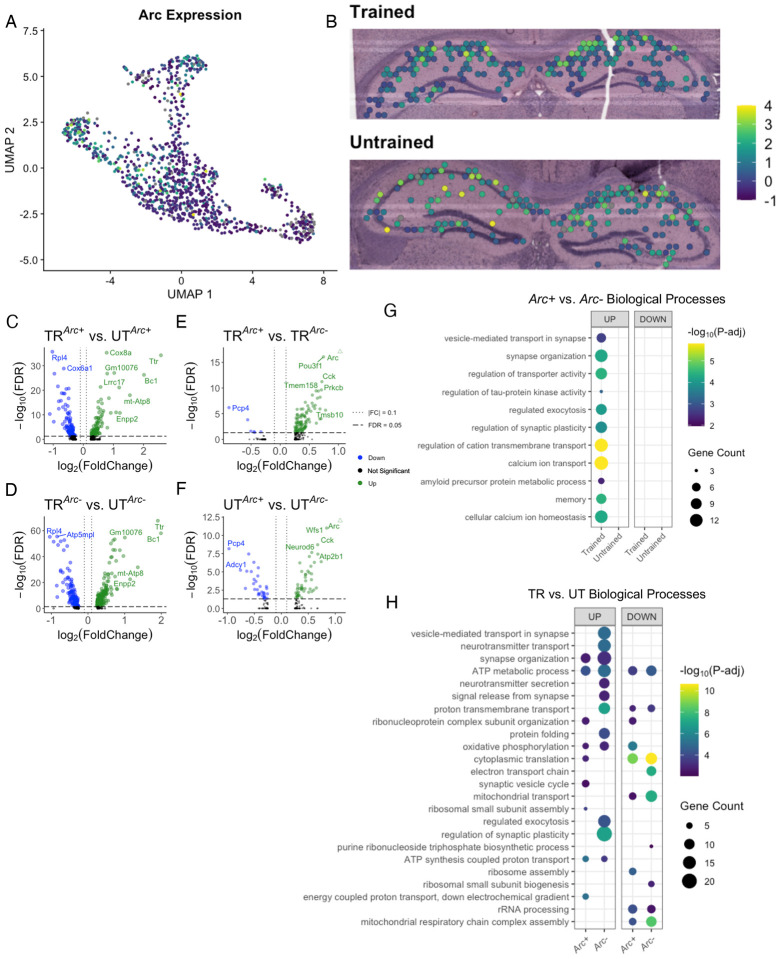
Arc-positive spots in the trained sample are enriched with genes involved in synaptic plasticity A,B) UMAP plot (left) and spatial transcriptomic maps of hippocampal spots (right) in the trained and untrained samples. Colors show normalized expression of the *Arc* gene in hippocampal cell layer spots. C,D) Volcano plots of differential gene expression of *Arc* positive (C) and negative (D) spots across behavioral conditions. E,F) Volcano plots of differential gene expression between *Arc* positive and negative spots within trained (E) and untrained (F) samples. G) Biological processes overrepresented amongst DEGs detected in the analysis of *Arc*-expressing spots within trained and untrained samples. Dot color reflects the statistical significance (−log_10_(FDR)) of the biological process enrichment. Dot size reflects the number of detected DEGs mapped to the genes involved in a given biological process. H) Biological processes overrepresented amongst DEGs detected in the analysis of *Arc*-expressing spots across trained and untrained samples. Dot color reflects the statistical significance (−log_10_(FDR)) of the biological process enrichment. Dot size reflects the number of detected DEGs mapped to the genes involved in a given biological process.

Our data suggest that differences in behavioral conditioning contributed to variations in the spatial distribution of synaptic plasticity related gene expression across the hippocampal network. Differences in behavioral conditioning affect the recruitment of Arc-expressing hippocampal neurons which form the memory-associated neuronal ensemble [60–63]. We studied the *Arc*-expressing spatial transcriptomics spots as a proxy for the putative IEG tagged memory-associated neuronal ensemble to assess changes in biological processes following APA memory recall. Out of the 542 spots in the hippocampus from the trained sample, 195 spots had detectable expression of *Arc* mRNA (*Arc+*). In the untrained sample, 169 out of 568 hippocampal spots were *Arc+* (Supplemental Figure 3G). In both samples, spots with the highest level of *Arc* expression were located in the CA1 cell layer (Figure 6A,B).

To test whether *Arc*-expressing neuronal ensembles exhibit changes in the expression of genes related to synaptic plasticity, we performed differential gene expression analyses between both: *Arc*+ spots across trained and untrained samples (TR^*Arc+*^ vs. UT^*Arc+*^), and between *Arc*+ and *Arc*− spots within each sample (TR^*Arc+*^ vs. TR^*Arc*−^ & UT^*Arc+*^ vs. UT^*Arc*−^) (Figure 6 C–F). Comparison of TR^*Arc+*^ vs. UT^*Arc+*^ spots detected 174 significant DEGs (85 upregulated and 89 downregulated genes), and comparison of TR^*Arc*−^ vs. UT^*Arc*−^ detected 353 significant DEGs (113 upregulated and 240 downregulated genes) (Figure 6 C,D). *Bc1* and *Rac1*, known for their involvement in learning and memory [64, 65], were found to be down- and upregulated, respectively, in the analysis across both *Arc*+ and *Arc*− spots. GO term enrichment analysis of upregulated DEGs detected across the TR^*Arc+*^ vs. UT^*Arc+*^ showed enrichment with biological processes related to synaptic signaling and protein translation (Figure 6 H & Supplemental Figure 3C,E). Upregulated DEGs detected across the TR^*Arc*−^ vs. UT^*Arc*−^ spots enriched for genes related to synaptic transport and plasticity. Conversely, across both TR^*Arc+*^ vs. UT^*Arc+*^ and TR^*Arc*−^ vs. UT^*Arc*−^ spots, functions related to energy balance and protein translation were downregulated. 101 DEGs (95 upregulated, 6 downregulated) were detected within TR^*Arc+*^ vs. TR^*Arc*−^, and 78 DEGs (47 upregulated, 31 downregulated) were detected within UT^*Arc+*^ vs. UT^*Arc*−^ (Figure 6 E,F). *Dkk3*, *Cck*, and *Neurod6*, all genes related to learning and memory, were detected in *Arc+* spots within the hippocampus from both the trained and untrained mice [66–68]. *Homer1*, *Sv2b*, and *Scn3b* were among the 17 significant DEGs detected in both the analysis of TR^*Arc+*^ vs. TR^*Arc*−^ spots and the bulk comparison of all subregions. While DEGs detected within UT^*Arc+*^ vs. UT^*Arc*−^ spots were not significantly enriched with genes involved in any biological processes, TR^*Arc+*^ vs. TR^*Arc*−^ spots were significantly enriched with upregulated DEGs involved in synaptic plasticity (Figure 6 G & Supplemental Figure 3D,F).

### IEG-expressing hippocampal spots comprise distinct gene expression profiles within trained and untrained samples

Subsets of the complete memory-associated neuronal ensemble can be marked through the expression of different IEGs [69, 70]. To evaluate the functional relationship between these subsets of the ensemble we correlated the expression of 22 known neuronal IEGs [71, 72] with the expression of *Arc*. Pairwise correlations of the expression of these IEGs identified *Egr1* and *c-Jun* as the IEGs most and least correlated with *Arc*, respectively (Supplemental Figure 3 A,B). The correlation coefficients for *Arc* and *Egr1* are r(21) = .598, p = 2.24 × 10^−56^ in the untrained sample and r(21) = .764, p = 5.45 × 10^−105^ in the trained sample. The correlation coefficients for *Arc* and *c-Jun* are r(21) = −.057, p = 0.17 in the untrained sample and r(21) = −.105, p = 0.014 in the trained sample. Because of this, we performed differential gene expression analyses on *Egr1* and *c-Jun* expressing spots across and within behavioral conditions.

**Figure 7.**
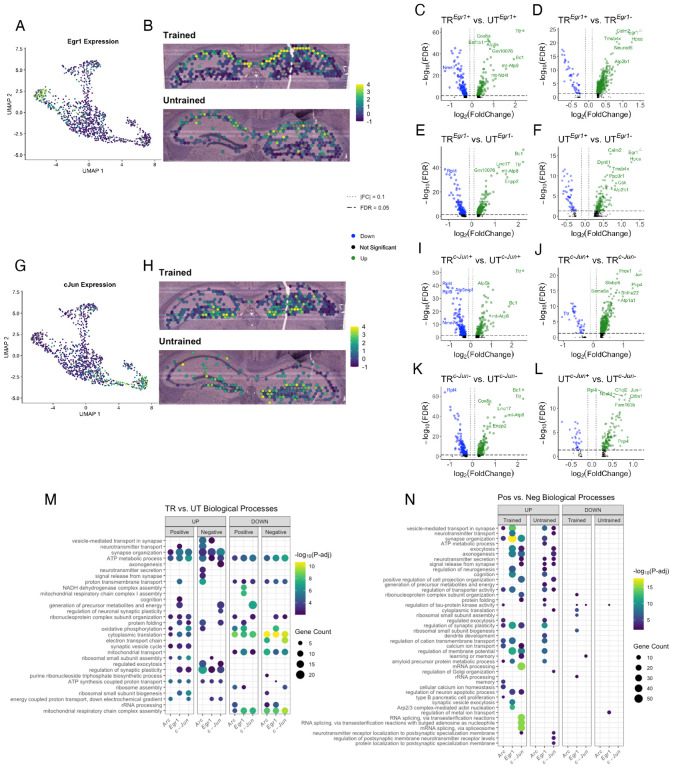
Spatial transcriptomics reveals distinct collections of biological processes enriched among *Arc*, *Egr1*, and *c-Jun*-expressing spots A,B) UMAP plot (left) and spatial transcriptomic maps (right) of hippocampal spots in the trained and untrained sample. Color reflects the normalized expression of Egr1 in the hippocampal cell layer spots. C,D,E,F) Volcano plots of differential gene expression of Egr1 positive (C) and negative (D) spots across behavioral conditions and within trained (E) and untrained (F) samples. G,H) UMAP plot (left) and spatial transcriptomic maps (right) of hippocampal spots in the trained and untrained samples. Color reflects normalized expression of c-Jun in the hippocampal cell layer spots. I,J,K,L) Volcano plots of differential gene expression of c-Jun positive (I) and negative (K) spots across behavioral conditions and within trained (J) and untrained (L) samples. M) Biological processes overrepresented amongst DEGs in Arc, Egr1, and c-Jun-expressing spots between trained and untrained samples. Dot color reflects statistical significance (−log10(FDR)) of biological process enrichment. Dot size reflects number of detected DEGs mapped to the genes involved in a given biological process. N) Biological processes overrepresented amongst DEGs in Arc, Egr1, and c-Jun-expressing spots across trained and untrained samples. Dot color reflects statistical significance (−log10(FDR)) of biological process enrichment. Dot size reflects the number of detected DEGs mapped to the genes involved in a given biological process.

In total, 380 out of 542 spots in the hippocampus of the trained animal and 256 out of 568 in the untrained were found to express detectable levels of *Egr1* (Supplemental Figure 3G). In both samples, spots with the highest level of *Egr1* expression were located predominantly in the CA1 cell layer, following the spatial distribution of *Arc* expression. For the *Egr1*-expressing spots, 555 DEGs (509 upregulated, 46 downregulated) were identified the trained animal, and 196 DEGs (150 upregulated, 46 downregulated) in the untrained animals (Figure 7 D,F). In total, we found detectable levels of *c-Jun* in 308 out of 542 spots in the hippocampal sample from the trained animal and 175 out of 568 in the untrained animal (Supplemental Figure 3G). In both samples, spots with the highest level of *c-Jun* expression were located predominantly in the DG granule cell layer. For the *c-Jun*-expressing spots, 480 DEGs (440 upregulated, 40 downregulated) were identified within the trained animal, and 166 DEGs (138 upregulated, 28 downregulated) within the untrained animal (Figure 7 J,L).

Due to the high degree of correlation between the expression of *Arc* and *Egr1*, and the low correlation between the expression of *Arc* and *c-Jun*, we predicted that *Arc* and *Egr1*-expressing spots would appear more functionally similar to one another than they would to *c-Jun*-expressing spots. However, GO enrichment analyses of all three IEG expressing spot populations across trained and untrained conditions showed similar sets of enriched biological processes (Figure 7M & Supplemental Figure 3C,E), namely those involved in gene expression and energy balance. In contrast to the within sample analyses of *Arc*+ spots showing overrepresentation of biological process only in the hippocampus of the trained mouse (Figure 6G), analyses of *Egr1*+ and *c-Jun*+ spots in the hippocampal samples of *both* trained and untrained mice revealed DEGs that were enriched in numerous biological processes. Biological processes enriched among DEGs in the *Egr1*+ spots within the hippocampus of trained and untrained mice were similar, and involved in synaptic plasticity and transmission (Figure 7N & Supplemental Figure 3C,E). Additionally, in the hippocampus of the trained mouse, *Egr1+* spots show downregulation of biological processes related to protein translation and ribosomal function. A distinct collection of biological processes were enriched among DEGs detected in analysis of *c-Jun*+ spots within the trained and untrained samples. Biological processes involved in axonal growth and neurotransmitter release were overrepresented in *c-Jun*+ spots in both the hippocampal sections from trained and untrained mice.

### RT-qPCR of DEGs detected in the overlap between bulk RNA sequenced hippocampal subregions

To further validate the spatial distribution of DEGs across the hippocampal network, we performed RT-qPCR comparisons of relative gene expression in micro-dissected DG, CA3, and CA1 subregions (6 trained and 4 untrained animals). To support the regionalized differential expression of the genes identified in the bulk RNA sequencing data, we measured the expression of *Kit*, *Sst*, *Cdh24* (identified in the DG, CA3, CA1 subregions, respectively). *Gapdh* expression was measured as an internal control. Expression of *Slc6a7* was also measured. *Slc6a7* was found to be upregulated in all regional analyses (Figure 3E, center sector). Relative gene expression demonstrate a significant upregulation of *Kit* in the DG of trained animals (p-adj = 0.024). *Sst* was also found to be upregulated in the DG (Supplemental Figure 4A). We found no significant differences for the measured genes in CA3 (Supplemental Figure 4B). In CA1, *Cdh24* is increased in trained compared to untrained samples although the difference did not reach statistical significance (Supplemental Figure 4C). And, *Slc6a7* was not increased in any subregion.

## DISCUSSION

In this study, we used a combination of bulk RNA sequencing and spatial transcriptomics to identify changes in gene expression within major subregions of the dorsal hippocampus and within IEG-expressing spots following APA memory recall. We hypothesized that forming a memory-associated neuronal ensemble following memory training and recall leads to the enrichment of unique biological processes across the dorsal hippocampus. To test this hypothesis, we used a combination of bulk RNA sequencing and spatial transcriptomics to identify changes in gene expression within each major subregion of the dorsal hippocampus and within IEG-expressing spots following memory recall. Vanrobaeys et al. used spatial transcriptomics to study consolidation of a spatial memory and found distinct yet overlapping transcriptomic signatures across hippocampal subregions, with the CA1 and DG exhibiting upregulation of genes related to transcriptional regulation and protein folding, respectively [73]. The findings from this study illustrate the importance exploring the spatial distribution of gene expression profiles when investigating the memory-associated neuronal ensemble and the functional changes therein.

### Regionalization of molecular mechanisms following memory recall

Different brain regions are recruited depending on the type of memory training an animal is exposed to [27, 74]. We found evidence supporting the regionalization of differential gene expression and overrepresentation of GO terms (corresponding to specific biological processes) across the dorsal hippocampus following spatial memory recall. Bulk RNA sequencing analyses between trained and untrained samples revealed significant similarities in the DEGs detected in CA1 and CA3 fields, but not between either of these subregions and the DG. Similarly, the biological processes overrepresented amongst DEGs in the CA1 and CA3 subregions shared more similarities than with those in the DG. Spatial transcriptomics also revealed regional differences in DEG detection and biological process overrepresentation. DEGs detected via spatial transcriptomics did not show considerable overlap compared with those detected via bulk RNA sequencing. Such differences might be accounted for by the technical differences between the two transcriptomic methodologies (addressed below).

Cells in the CA1 and CA3 subregions share similar morphologies and ontology as compared to the dentate gyrus [75–77], which could account for our regionalized findings in the bulk RNA sequencing study. During memory recall, the functional coupling of the CA3 and CA1 subregions [78, 79] is also reflected by the gene expression changes observed in this study. Our data show that the spots in the CA3 and CA1 subregions have upregulated biological processes involved in post synaptic machinery and synaptic plasticity, while the spots in the DG subregion have upregulated processes involved in protein expression and energy balance, and have downregulated processes involved in synaptic transmission. The cellular and molecular modifications occurring during memory recall are known to include: 1) increases in neurogenesis and protein synthesis in the DG [80–83] and 2) increased synaptic efficacy (e.g., LTP) across the Schaffer collateral between the CA3 and CA1 [84, 85]. The overrepresentation of GO terms involved in ATP metabolism and energy balance in the DG could indicate an understudied role of the differential regulation of metabolic pathways in support of memory storage and recall in the hippocampus [86, 87]

Circuitry in the hippocampus is differentially recruited depending on the phase, cognitive demand, and valance of a memory[88–90]. We believe that the functional changes observed during the recall phase of memory are one snapshot of the spatio-temporal modifications occurring over the life of a memory in the brain. More studies are required to evaluate sequential changes in regionalized hippocampal function with memory over time.

### Technical considerations of using spatial transcriptomics to study memory-associated neuronal ensembles

We utilized a combination of bulk RNA-seq and spatial transcriptomics to measure the gene expression changes in the brain following memory recall. Both methodologies provided unique utility and insight into the transcriptomic changes in the brain following memory and have proven to be highly compatible [73, 91]. We observed strong regionalized differences between training conditions, despite the fact that the changes we observed did not align between the two transcriptomic techniques. This discrepancy can be accounted for by some of the chemical and technical differences between the two methodologies. First, 10x’s Visium spatial transcriptomic platform permits a granular resolution of neuronal systems with its 55μm diameter capture spots [92]. The resolution of this platform allows us to analyze gene expression differences between specifically annotated populations of neurons in the cell layers of each hippocampal subregion, rather than the entirety of the bulk section. Second, relative to bulk sequencing, 10x’s Visium spatial transcriptomics requires more tissue processing before RNA extraction and has lower RNA detection sensitivity [93]. Consequently, some DEGs with lower levels of expression in our bulk study may have gone undetected in the spatial transcriptomic study, thus skewing which genes were profiled. Our study is amongst the first to examine gene expression changes within individual cell populations across a spatially distributed neuronal network. The exploratory nature of the present study, together with more accessible and higher resolution spatial transcriptomic methods, is expected to develop into deeper characterizations of the mechanistic architecture of memory.

### *Arc*-expressing spots are enriched with genes involved in synaptic plasticity

Using spatial transcriptomics, we were able to detect the spatial distribution of immediate early gene expression in the dorsal hippocampus. Spatial transcriptomic spots sample regions 55μm in diameter, which can contain more than a dozen cell bodies (depending on the brain region). We inferred that groups of IEG-expressing memory-associated neurons would be sampled by these spots, which we predicted would be enriched with genes related to synaptic plasticity. To accomplish this, we targeted the expression windows of both immediate early genes and late response genes by sacrificing mice following the retention test during the peak of IEG expression and the beginning of late response gene expression [94, 95].

Data from the *Arc*-expressing spots highlight the distinct gene expression profile of TR^*Arc+*^ spots. Only the upregulated DEGs detected in the analysis of TR^*Arc+*^ vs. TR^*Arc*−^ were enriched with any biological process. This pattern of enrichment indicates both that TR^*Arc+*^ neurons are functionally distinct from the surrounding TR^*Arc*−^ cells and the UT^Arc+^ cells. Specifically, TR^*Arc+*^ cells are defined by an upregulation and enrichment of biological processes related to synaptic plasticity, a number of which were also upregulated and enriched in the bulk differential expression analyses of all hippocampal regions combined. These data suggest that the functional changes induced in the *Arc*-expressing spots might substantially contribute to the differences observed between training conditions in the analysis of bulk hippocampal subregions. We also detected DEGs and biological processes between the TR^*Arc+*^ vs. UT^*Arc+*^ spots. In addition to biological processes related to synaptic signaling, an abundance of biological processes related to energy metabolism and protein translation –ATP metabolic process, ATP synthesis coupled proton transport and cytoplasmic translation – were enriched in the TR^*Arc+*^ vs. UT^*Arc+*^ comparison. It is not yet known if the functional changes that distinguish cell in a memory-associated neuronal ensemble from the surrounding cells are the same functional changes that distinguish these cells across training conditions. Our data suggests that there are two defining features of the *Arc+* spots. The first is the upregulation of synaptic plasticity mechanisms when compared to *Arc−* spots. This finding is supported by a recent study that found synaptic plasticity mechanisms as a defining feature of a neuron in a memory-associated neuronal ensemble [96]. The second is the increased recruitment of cellular machinery involved in energy production when compared to the same spots in the hippocampus of the untrained animal. This result is supported by increases in energy production and consumption during periods of high cognitive demand on neuronal systems [97] In the case of the APA, the trained avoidance behavior is considered higher cognitive demand than the untrained context habituation behavior [40, 98]. Arc+ spatial spots in the trained animal are different from Arc− spots in the same animal and Arc+ spots in the untrained animal.

### *Egr1*- and *c-Jun*-expressing spots exhibit distinct transcriptomic profiles

The neuronal ensemble tagged by Arc is well studied for its role in memory storage and maintenance [99], but the *Arc*-tagged ensemble encompasses only a subset of the neurons that were active during a memory experience[69, 99, 100]. Subpopulations of this memory-associated neuronal ensemble across brain systems might encode for multiple memory traces relevant to a behavioral experience [1, 27, 101, 102]. Separate subpopulations of these neuronal ensembles are tagged by at least one of the two dozen IEGs, the expression of which is linked to one of the many patterns of strong neuronal activity elicited during a memory experience [27, 71, 103–105]. With spatial transcriptomics, we assessed gene expression profiles of IEG positive spots in the dorsal hippocampus after the retention test in trained and untrained animals. We expanded our investigation of the gene expression profile of the memory-associated neuronal ensemble to the spots tagged by the IEGs *Egr1* and *c-Jun*. The expression of *Egr1* was most correlated with the expression of *Arc*, while *c-Jun* was the least correlated.

Sparing some overlap, the populations of *Arc+, Egr1+*, and *c-Jun+* spots within both samples were each enriched with genes involved in distinct collections of biological processes. DEGs detected in the analysis of *Egr1+* and *c-Jun+* spots in *both* samples from trained and untrained mice were enriched in numerous biological processes. However, DEGs detected in the analysis of *Arc*+ spots within *only* the trained sample were enriched with any biological processes, namely those related to synaptic plasticity and synaptic function. The *Egr1+* spots within both training conditions detected upregulated genes involved in biological processes related to synaptic plasticity and synaptic transmission. *c-Jun*+ spots within both training conditions detected upregulated genes involved in biological processes related to axonal growth and neurotransmitter release. Unlike the *Arc*-expressing spots in the trained sample, biological process were enriched among downregulated DEGs detected *Egr1-* and *c-Jun*-expressing spots the trained sample. Additionally, the spatial distribution of *Arc* and *Egr1* shows high levels of expression in the CA1 cell-layer of trained and untrained samples, while the expression of *c-Jun* is high in the DG. Expression of each IEG might be linked to particular patterns of neuronal activation [106–108] [109] and output circuit function [89], highlighting the functional and spatial diversity amongst recruited cells within memory associated neuronal ensembles. *Egr1* and *c-Jun* expression are associated with fear memory and acute stress, respectively [110–112].

Perhaps, the differences observed amongst the *Egr1+* and *c-Jun+* spots may not be specific to the behavior of trained active place avoidance behavior, but rather, sensitive to the shared emotional, temporal and contextual components inherit to both long term memory experiences of trained and untrained animals in the APA apparatus.

In contrast to the diversity of biological processes enriched among the DEGs between IEG-expressing spots *within* each training condition, we found very similar collections of biological processes in the GO enrichment analysis of IEG+ spots *across* training conditions. These differentially regulated processes involved in energy production suggest that the training experience induces a greater energy demand on the memory associated neuronal ensemble, relative to the untrained experience. These data further highlight the need for greater energy metabolism in higher cognitive demand memory experience, like that of training in the APA [87, 97].

### Investigating memory across multiple scales in the brain

In summary, we performed an investigation of the transcriptomic profile of spatial memory recall in the hippocampus using a combination of bulk RNA sequencing and spatial transcriptomics. We found a regionalized pattern of gene expression with the CA1 and CA3 subregions being enriched with genes involved in synaptic plasticity and transmission, while the DG subregion was enriched with genes involved in protein expression and energy production. We also discovered a unique transcriptomic profile of *Arc* expressing spots in the dorsal hippocampus of trained mice enriched for upregulated genes involved synaptic plasticity and transmission. Additionally, the transcriptomic profile of these *Arc* expression spots differed from those other IEG expressing spots (i.e. *Egr1* and *c-Jun*).

This is amongst the first steps toward bridging our understanding of memory *across* scales ranging from systems to cells to molecules. In this study, we investigate the spatial distribution of memory-associated molecular function following the reactivation of a memory experience. Through the joint investigation of the effectors of memory – their neuronal identities, spatial location and functional changes – we open the opportunity for future studies that seek to understand how memories are stored in the brain and how they change over time. Through similar transcriptomic approaches that focus on space as a biologically relevant variable, we will begin to map the distribution of memory-associated neuronal ensembles and their respective gene expression changes as they undulate across the hippocampal network over the lifetime of particular memory experiences.

## METHODS

### Animals

A total of 18 adult ArcCreERT2::eYFPflx mice [113] with C57BL/6 genetic background aged 3–4 months were used in this study (Bulk RNA sequencing – 3 male and 3 female, spatial transcriptomics – 2 male, RT-qPCR – 10 male). Mice were bred in-house at the SUNY Downstate Health Sciences University vivarium (Brooklyn, NY, USA). Mice were housed in groups of two to five until the start of the experiment, at which point they were single-housed in shoebox cages in a sound attenuation cubicle (Med Associates). For every experimental run, a minimum of two mice —each undergoing the same behavioral conditioning— were simultaneously housed in the sound attenuation cubicle. Ad libitum food and water was provided. Mice were randomly assigned to behavioral cohorts before the start of the experiment. Mice were handled daily for 3 days prior to the start of the experiment to reduce anxiety and improve voluntary approach. All animal procedures proposed are approved by, and will be performed following, the Institutional Animal Care and Use Committee guidelines at SUNY Downstate Health Sciences University.

### Behavior

All procedures were performed in compliance with the Institutional Animal Care and Use Committee of the State University of New York, Downstate Health Sciences University. ArcCreERT2::eYFPflx male mice were trained in a hippocampus-dependent two-frame active place avoidance task. The place avoidance system consisted of a 40-cm diameter arena with a parallel rod floor that could rotate at 1 rpm. The position of the animal was tracked using PC-based software (Tracker, Bio-Signal Group Corp., Brooklyn, NY) that analyzed 30-Hz digital video images from an overhead camera. Mice in the trained condition learned the “Room+Arena−” task variant. Place avoidance of a 60° zone was reinforced by a constant current foot shock (60 Hz, 500ms, 0.2mA) that was scrambled (5-poles) across pairs of the floor rods. Rotation of the arena would carry the mouse into the shock zone unless the animal actively avoided the zone. Entering the shock zone for more than 500 ms triggered shock. Additional shocks occurred every 1.5 seconds until the animal left the shock zone. Measures of place avoidance were computed by TrackAnalysis software (Bio-Signal Group Corp., Brooklyn, NY). On day 1, mice received a 30-min trial with the shock off to habituate to the rotating arena. Across the next two days the animals experienced 4 training trials, with 2 30-minute trials a day with the activated shock with a 40 min inter-trial-interval.. Control (untrained mice) experienced identical training conditions, except for the shock always being off.

Memory performance was assessed 24 hr after the final training session in a 10 minute retention test with the shock off.

### Microdissected bulk RNA sequencing

60 minutes after retention test, mice were euthanized and brains were extracted, washed in ice washed in ice cold artificial cerebrospinal fluid, blocked, and mounted on a vibratome stage. Hippocampal subregions (DG, CA3, CA1) were microdissected from 400 μm thick coronal live tissue sections. Subregions from the dorsal hippocampus were extracted and microdissected using microsurgical tools. Microdissected pieces of tissue from each animal were pooled by subregion in 500 μL of prechilled TRIzol in a 1.5mL microcentrifuge tubes and stored at −80°C. Total RNA was extracted using the Zymo RNA mini prep kit (Zymo Research, Irvine, CA, United States) according to the manufacturer’s protocol. RNA quality was assessed on an Agilent 2200 TapeStation (Agilent Technologies, Palo Alto, CA, United States). Samples with a RIN greater than 7 were processed for library preparation. For library preparation NEBnext ULTRA II RNA Library Preparation Kit for Illumina (New England Biolabs, Ipswich, MA, United States) was used to: enrich mRNA from total RNA through rRNA depletion, fragment mRNA into short fragments using fragmentation buffer, reverse transcribe mRNA into cDNA using random primers, synthesize second-strand cDNA with DNA polymerase I, RNase H, dNTP, and buffer. Double stranded cDNA fragments were preapered into sequenceable libraries through multiple steps of purification using SPRIselect beads, end repair, A-tailing and adaptor ligation. The ligated library was size selected using SPRIselect beads, PCR amplified and sequenced using the Illumina Novaseq6000.

### Spatial transcriptomics

60 minutes after retention test, mice were euthanized and brains were extracted and immediately prepared for snap freezing in cuvettes of Tissue-Tek Optimal Cutting Temperature compound (Sakura Finetek USA, Torrance, CA, United States) floating in a bath of methyl-butane chilled by liquid nitrogen. Brain blocks were cryosectioned at −20°C [114] and 10 μm thick coronal sections containing the dorsal portion of the hippocampus were obtained (bregma −1.7 and −2.2 mm [115]). Collected tissue sections are trimmed to fit within the 5 mm × 5 mm capture area on the Visium gene expression slide. Sections were mounted on a prechilled Visium gene expression slide or a Tissue Optimization slide. A single tissue block was use to collect all tissue sections mounted on the tissue optimization slide. One section of tissue per sample was mounted in the capture areas of the Visium gene expression slide.

Tissue sections were fixed in chilled methanol and stained according to the Visium Spatial Gene Expression User Guide [116] or Visium Spatial Tissue Optimization User Guide [117].

Tissues were permeabilized on the gene expression slide for 18 minutes which was selected from the results of our tissue optimization experiment. Images were taken according to the Visium Spatial Gene Expression Imaging Guidelines[118]. Brightfield histology images for the Visium Tissue Optimization and Gene Expression slides were taken using the Leica Aperio CS2 Slide Scanner at 20x magnification. Tissue optimization fluorescent images were taken on a Zeiss LSM 800 (555nm LED,75% intensity, and 200ms exposure).

Tissue mRNA was extracted and libraries were prepared following the Visium Spatial Gene Expression User Guide [116]. They were loaded at 300 pM and sequenced on a NovaSeq 6000 System (Illumina) using a NovaSeq S4 Reagent Kit (200 cycles, catalog no. 20027466, Illumina), at a sequencing depth of approximately 250–400 10 × 106 read-pairs per sample. Sequencing was performed using the following read protocol: read 1: 28 cycles; i7 index read: 10 cycles; i5 index read: 10 cycles; and read 2: 91 cycles.

### RNA sequencing data analysis

Sequencing reads are processed at the Downstate Human Genomics Advanced Computing Facility. Sequencing fastq files were demultiplexed with salmon, filtered and mapped to the mouse MM10 reference genome [119] using STAR Aligner [120] and transcript reads were quantified for each sample.

### Bulk RNA sequencing analyses

#### Differential Gene Expression

Differential expression analysis was performed through DESeq2 on RNA counts between trained and untrained sample groups. Genes with an of FDR below 0.05 were considered differentially expressed genes/transcripts.

#### PCA

We ran 4 PCA analyses on normalized counts for samples. One analysis on all the the subregions combined and 1 one the samples from each hippocampal subregion, 3 total.

#### Heatmap

Genewise Z-scores were calculated for the counts of each gene in each sample. Z-scores are plotted as a heat map, where each row is a gene, and each column is a sample. Rows and columns are then sorted in a dendrogram using Euclidean distance as a measurement for similarity between any two given columns or rows.

#### Gene Ontology Enrichment Analysis

We used clusterProfiler’s Overrepresentation analysis for GO terms [121] which uses the hypergeometric test to calculate the enrichment Biological Processes, Cellular Components and Molecular Functions amongst a list of differentially expressed genes (FDR < 0.05) against a background of all detected genes. GO terms enriched with an FDR < 0.01 were considered significant for our analyses. This analysis was also used for DEGs detected in the spatial transcriptomic analyses. Dot plot figures show the top 10 GO terms ordered by significance for each analysis plotted in the figured.

#### Venn Diagram

Vennplex software[122]was utilized to plot overlaps in the differentially expressed gene lists from our regional differential gene expression analyses in a venn diagram format. This software, stratifies gene lists by up- and down-regulated genes to display how many genes in overlapping sectors are upregulated, downregulated or counterregulated (same gene with opposite effect sizes in two regions).

#### Gene overlap statistics

The R-language package “SuperExactTest” [123] was used to statistically test the overlap of gene names between regional lists of DEGs. The package uses the hypergeometric Fisher’s Exact test, against a background of all genes successfully compared in each differential gene expression analysis. The software package also outputs an upset-style plot to visualize degree of overlap between lists of DEGs and the level of significance of the overlap.

### Spatial transcriptomics analyses

Reads from each sample were quantified and alighted to a predetermined loci or capture spots on the histological image of each sample using 10x’s space ranger software which references the fiducial frame surrounding each capture area (10x Genomics Space Ranger 1.1.0).

Gene expression data were analyzed using the software package Seurat in the R coding environment [124]. Spatial gene expression data were cropped to only include the capture spots within the dorsal hippocampus. Data from each sample were then normalized, transformed and integrated with one another using the default tools in Seurat. The integrated data set were further normalized and clustered in PCA space. Clustered data can be visualized through UMAP projections of the capture spots or in the 2D space of the tissue section. Cell type annotation was performed by integrating our data with a random subset of 10,000 hippocampal cells from the Allen Brain Atlas Cortex and Hippocampus Single Cell Taxonomy[56]. Predicted cell-type identities were calculated and optimized based on gene expression similarity and anatomical location.

#### Differential Gene Expression

Capture spots were grouped along two different criteria to calculate differential gene expression in our samples. First, capture spots belonging to the anatomical cell layers of the Dentate Gyrus, CA3 and CA1 regions were compared across training conditions together (All Regions) and separately (region-wise). Next, within each training condition, hippocampal capture spots were separated into two groups based on the detectable (>0) expression of the immediate early gene (e.g. *Arc+* and *Arc-* groups). IEG expressing and not expressing groups of spots were compared both within training conditions and across training conditions. Differential gene expression was calculated using the Wilcoxon rank sum test (the default method for Seurat). Genes with an of FDR below 0.05 were considered differentially expressed genes/transcripts.

#### Pairwise Correlations

Counts data for 23 known IEGs were extracted from hippocampal cell layer capture spots. In each sample, Pearson’s correlation coefficients were calculated pairwise for each IEG using the base statistics in R-language. P values for Pearson’s correlations were calculated for IEG’s using the base R-language correlation test.

### RT-qPCR

Samples were extracted similarly to as described for bulk sample preparation to extract the dorsal hippocampal subregions CA1,CA3, and DG from coronal sections of brain tissue. RNA extraction using guanidinium-phenol-cholorform extraction with ethanol precipitation was performed and quantified using spectrophotometry. cDNA was reverse transcribed from sample bulk RNA using Thermo Fisher’s SuperScript IV Kit (Thermo Fisher Scientific, Waltham, MA, United States). Relative gene expression was quantified using the SYBR green master mix system [125]. 10 μL reactions were prepared in the wells of a 384 well plate, hippocampal regions were each prepared on separated plates. Each well contained 2.5ng of template cDNA and 0.5 μM each of forward and reverse primers for one gene per well for a total reaction volume of 10μL (sequences and melting temperatures can be found in table 1). Each gene was loaded in triplicate in each plate to account for pipetting errors. Plates were incubated in the Bio-Rad CFX384 Real-Time PCR Detection System (Bio-Rad Life Sciences, Hercules, CA, United States) using the following schedule: 50°C for 5 min (1 cycle), 95°C for 10 min (1 cycle), 60°C for 1 min (1 cycle), and 95°C for 15 s followed by 60°C for 1 min (40 cycles).

## Supplementary Material

1

## Figures and Tables

**Table 1.** RT-qPCR primer sequences

Gene	Direction	Sequence	Tm (°C)
Gahpdh	F	AGG TCG GTG TGA ACG GAT TG	57.47
	R	TGT AGA CCA TGT AGT TGA GTC A	52.84
Sst	F	ACC GGG AAA CAG GAA CTG G	57.44
	R	TTG CTG GGT TCG AGT TGG C	58.45
Kit	F	GCC ACG TCT CAG CCA TCT G	58.51
	R	GTC GCC AGC TTC AAC TAT TAA CT	55.14
Cdh24	F	AGC CTG TCC TTA TTG GCA AGC	57.92
	R	TGG GCC AGC AGC ACA TAT TG	58.33
Slc6a7	F	ACC TGG ATG TAG ACT TCG CAG	56.47
	R	CGC CAG ACA TTT CCC AAG C	56.97

Following 40 cycles of amplification wells were heated in a gradient from 55oC to 90oC to build dissociation curves of primers and their transcripts to assess primer efficiency and specificity. Ct values for each reaction are then automatically determined (roughly 20% of the plateau value). Data were analyzed for relative gene expression quantification using the ΔΔCt method, using the expression of *Gapdh* as the internal control. Relative expression values were normalized to the expression value of the untrained group and Student’s t-test were performed on each primer target across training conditions using the ΔCt value for each comparison. Benjamini-Hochberg correction was performed to adjust p-values for multiple hypothesis testing.
