# Supplementary material for: Spatially Resolved Transcriptomic Signatures of Hippocampal Subregions and Arc-Expressing Ensembles in Active Place Avoidance Memory
Source: bioRxiv. 2024 Feb 16:2023.12.30.573225. Originally published 2024 Jan 1. Preprint. [Version 2] doi: 10.1101/2023.12.30.573225 (PMC10802250; doi:10.1101/2023.12.30.573225)
Supplement: Supplement 1 [file media-1.pdf]

1 SUPPLEMENTAL FIGURES

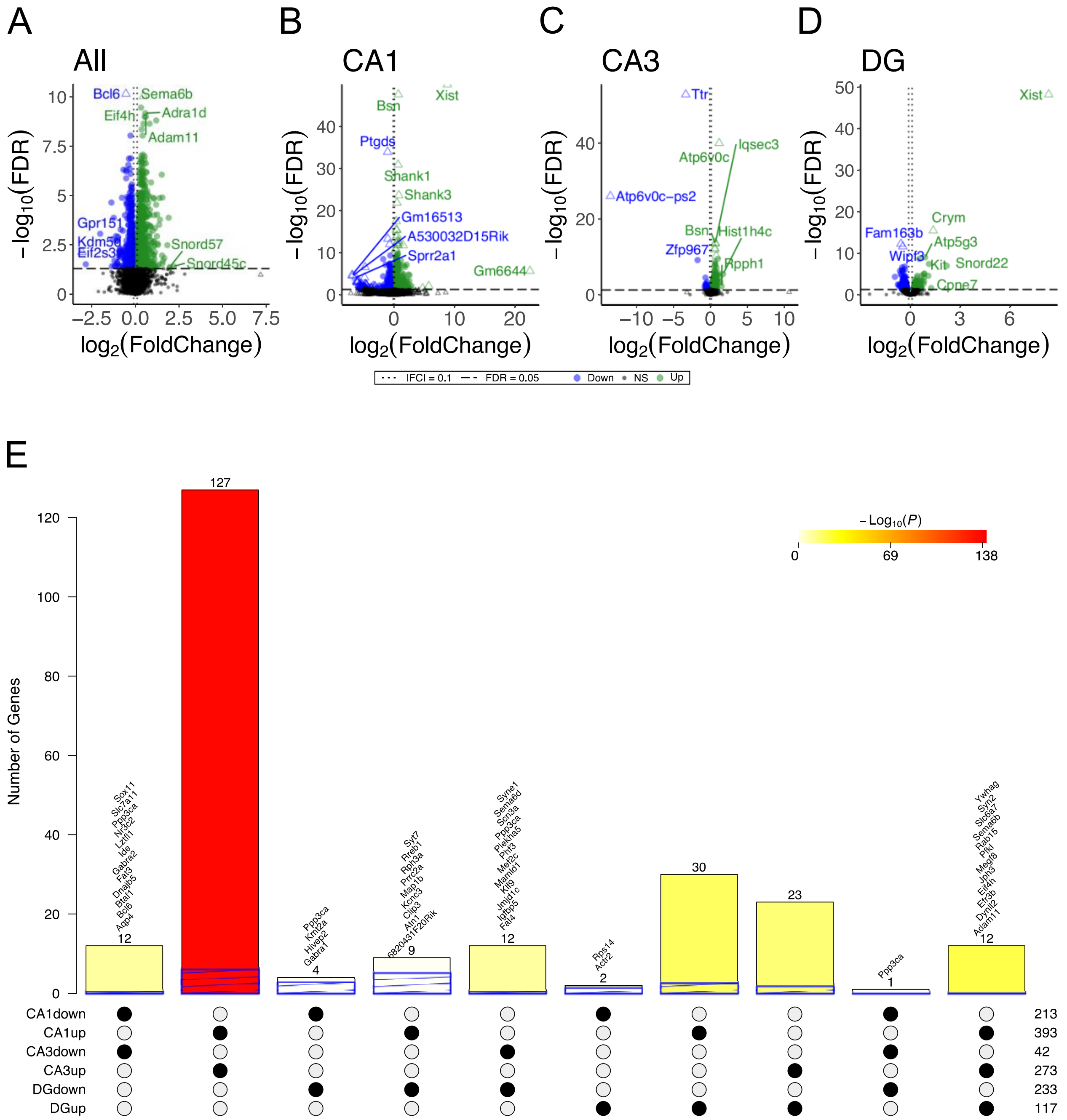

Supplemental Figure 1. Trained CA1 and CA3 subregions express similar DEGs and postsynaptic cellular component GO terms. **(A-D)** Volcano plot of DEGs between trained and untrained CA1, CA3 and DG samples, combined and individually. Triangular points mark data out of the frame of the main text figure. **(E)** Upset plot of overlaps in regional DEGs demonstrates greatest similarity between CA1 and CA3 samples. DEGs from each regional analysis were stratified by direction of fold change. In the upset plot, each bar represents the

number of genes in an intersection of lists of DEGs. The intersecting lists is represented by the dots underneath the bar. The hashed line on each bar represents the expected intersection of two identically sized random lists. Overlaps were tested with Fisher's exact test using the list of all expressed genes as a background. The color of the bar represents the $-\log_{10}(P)$ for the BH corrected P-value of each intersection.

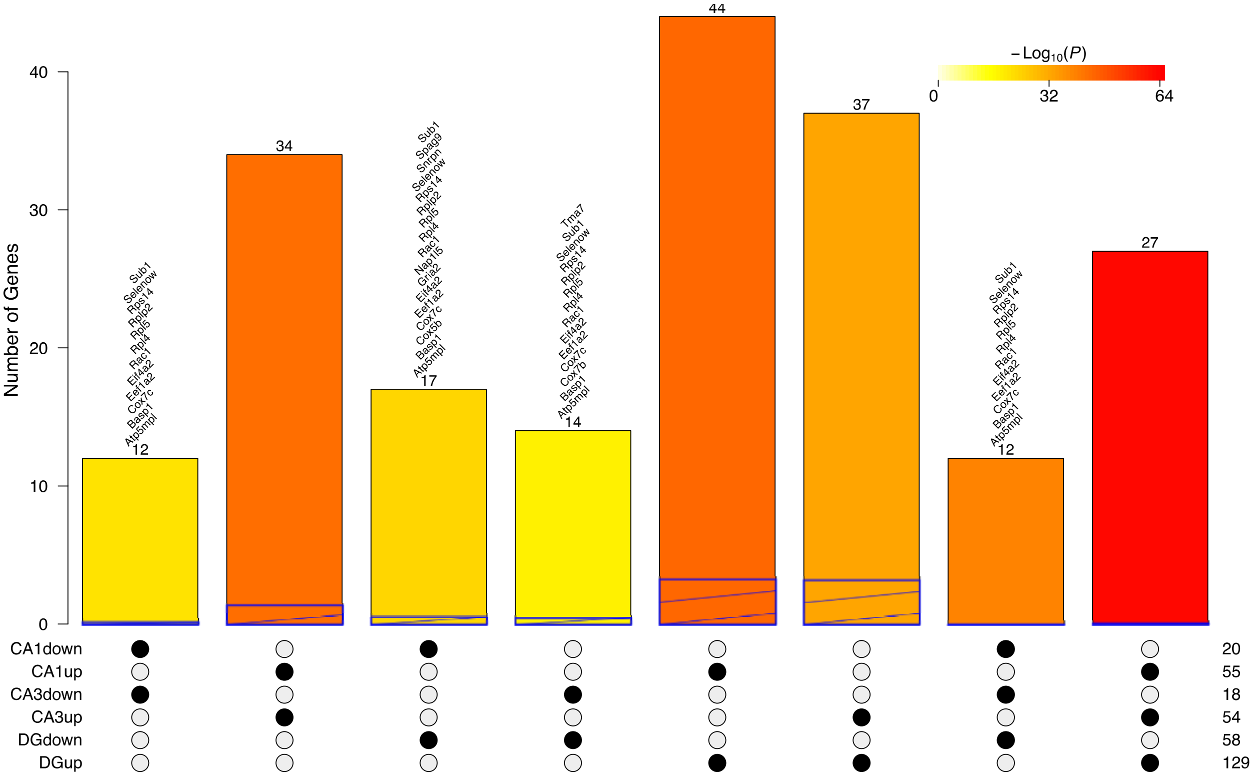

Supplemental Figure 2. Upset plot of overlaps in regional DEGs demonstrates greatest similarity between CA1 and DG subregions. DEGs from each regional analysis were stratified by direction of fold change. In the upset plot, each bar represents the number of genes in an intersection of lists of DEGs. The intersecting lists is represented by the dots underneath the bar. The hashed line on each bar represents the expected intersection of two identically sized random lists. Overlaps were tested with Fisher's exact test using the list of all expressed genes as a background. The color of the bar represents the -$\log_{10}$(P) for the BH corrected P-value of each intersection.

A

B

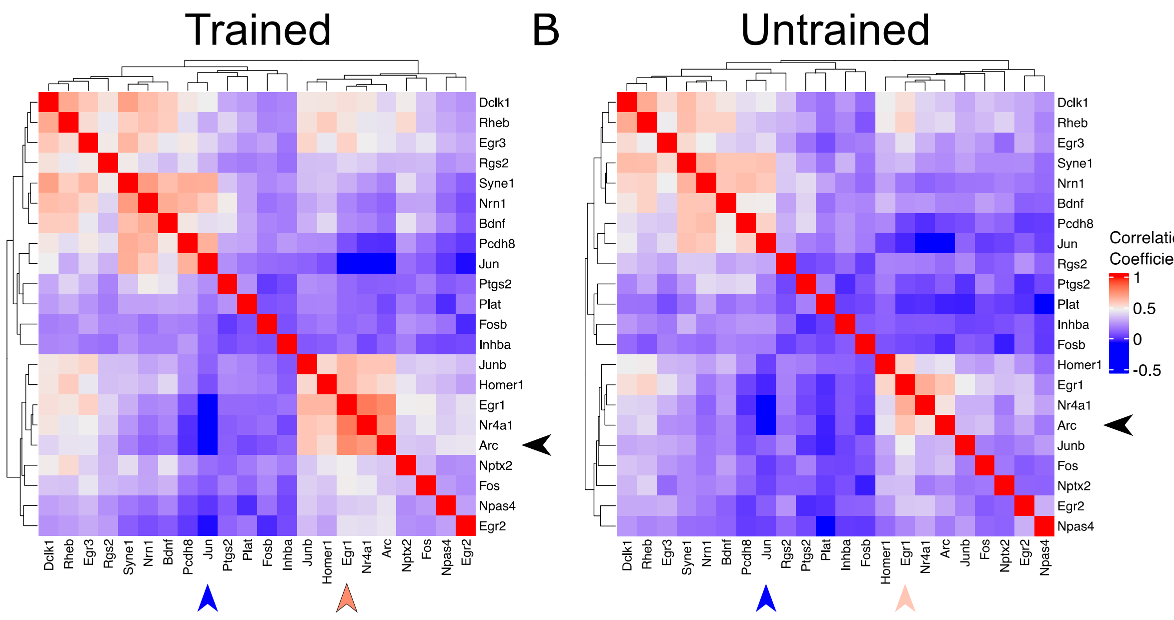

C

Cohort	IEG	All Spots	Cell Layers	CA1	CA3	DG
Trained	Arc	195/542	56/146	34/49	20/43	2/54
	Egr1	380/542	130/146	48/49	41/43	41/54
	c-Jun	308/542	106/146	22/49	32/43	52/54
Untrained	Arc	169/568	58/155	34/51	19/42	5/62
	Egr1	256/568	113/155	51/51	33/42	29/62
	c-Jun	175/568	83/155	12/51	18/42	53/62

D

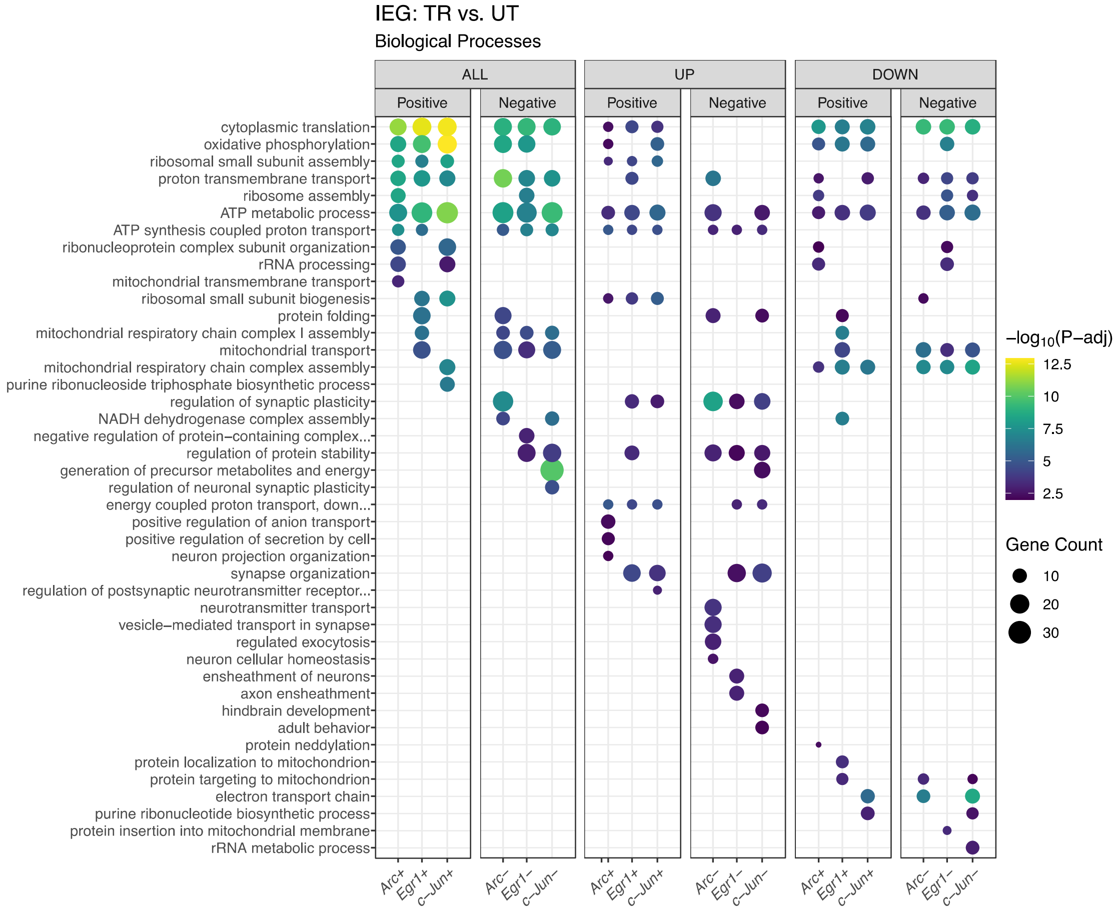

Supplemental Figure 3. The expression of the IEGs *Egr1* and *c-Jun* are most and least correlated with the expression of *Arc*, respectively. **(A, B)** Heatmaps reflecting the pairwise Pearson correlation coefficients between IEGs detected in the Trained **(A)** and Untrained **(B)** sample. Pearson correlation between *Arc* and *Egr1* or *c-Jun* are marked by the caret on the x-axis colored to reflect the value of the correlation coefficient. The correlation coefficients for *Arc* and *Egr1* are $r(21) = .598$, $p = 2.24 \times 10^{-56}$ in the untrained sample and $r(21) = .764$, $p = 5.45 \times 10^{-105}$ in the trained sample. The correlation coefficients for *Arc* and *c-Jun* are $r(21) = -.057$, $p = 0.17$ in the untrained sample and $r(21) = -.105$, $p = 0.014$ in the trained sample. **(C)** Table total number of IEG expressing spots relative to the total number of hippocampal spots for each area. **(D)** Biological processes overrepresented in the analysis of IEG-expressing spots across trained and untrained samples amongst all DEGs (left) and stratified by up- (middle) and down-regulated (right) DEGs. Dot color reflects the statistical significance ($-\log_{10}$(FDR)) of the biological process enrichment. Dot size reflects the number of detected DEGs mapping to the gene belonging to a given biological process.

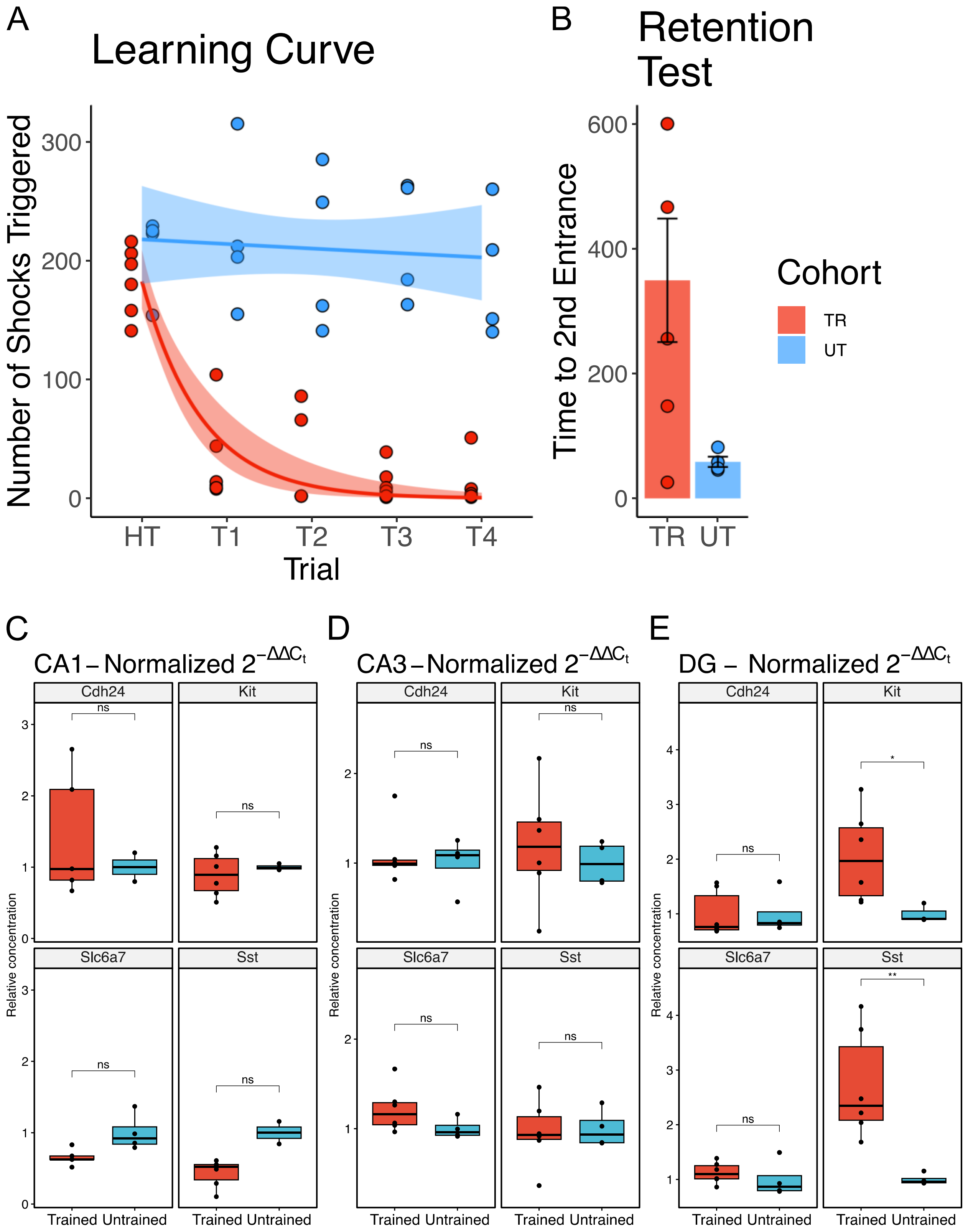
A
Learning Curve
Number of Shocks Triggered
0
100
200
300
HT
T1
T2
T3
T4
Trial
B
Retention Test
Time to 2nd Entrance
0
200
400
600
TR
UT
Cohort
TR
UT
C
CA1 – Normalized 2^-ΔΔCt
D
CA3 – Normalized 2^-ΔΔCt
E
DG – Normalized 2^-ΔΔCt
Cdh24
Kit
Slc6a7
Sst
ns
*
**
Relative concentration
Trained Untrained

Supplemental Figure 4. Relative expression of select DEGs through RT-qPCR. **(A,B)** RT-qPCR was performed on samples collected from animals trained under conditions identical to those used for bulk RNA sequencing or spatial transcriptomics. **(A)** Training performance measured as number of shocks triggered. Trained mice learned to avoid the location of the shock zone indicated by the decreased number of shocked triggered. Exponential fit learning curves included with shaded error bars (SE). **(B)** Memory performance measured as time to second entrance in the retention test trial. Trained mice demonstrated higher time to second entrance during the retention test. Trained mean 349s ± 98.9 (SE), Untrained mean 58.5s ± 8.23 (SE). **(C-E)** Average normalized relative expression ($2^{-\Delta\Delta Ct}$) of genes in trained samples (red) as compared to untrained samples (blue) in CA1 **(C)**, CA3 **(D),** and DG **(E)** subregions. n= 6 trained and n=4 untrained animals. Student t-test was performed between ΔCt values from trained and untrained samples for each gene.
