# Supplementary material for: Spatially Resolved Transcriptomic Signatures of Hippocampal Subregions and Arc-Expressing Ensembles in Active Place Avoidance Memory
Source: bioRxiv. 2024 Jan 1:2023.12.30.573225. Preprint. [Version 1] doi: 10.1101/2023.12.30.573225 (PMC10802250; doi:10.1101/2023.12.30.573225)
Supplement: Supplementary file 1 [file NIHPP2023.12.30.573225V1-supplement-1.pdf]

bioRxiv preprint doi: https://doi.org/10.1101/2023.12.30.573225; this version posted January 1, 2024. The copyright holder for this preprint (which was not certified by peer review) is the author/funder, who has granted bioRxiv a license to display the preprint in perpetuity. It is made available under aCC-BY 4.0 International license.

SUPPLEMENTAL FIGURES

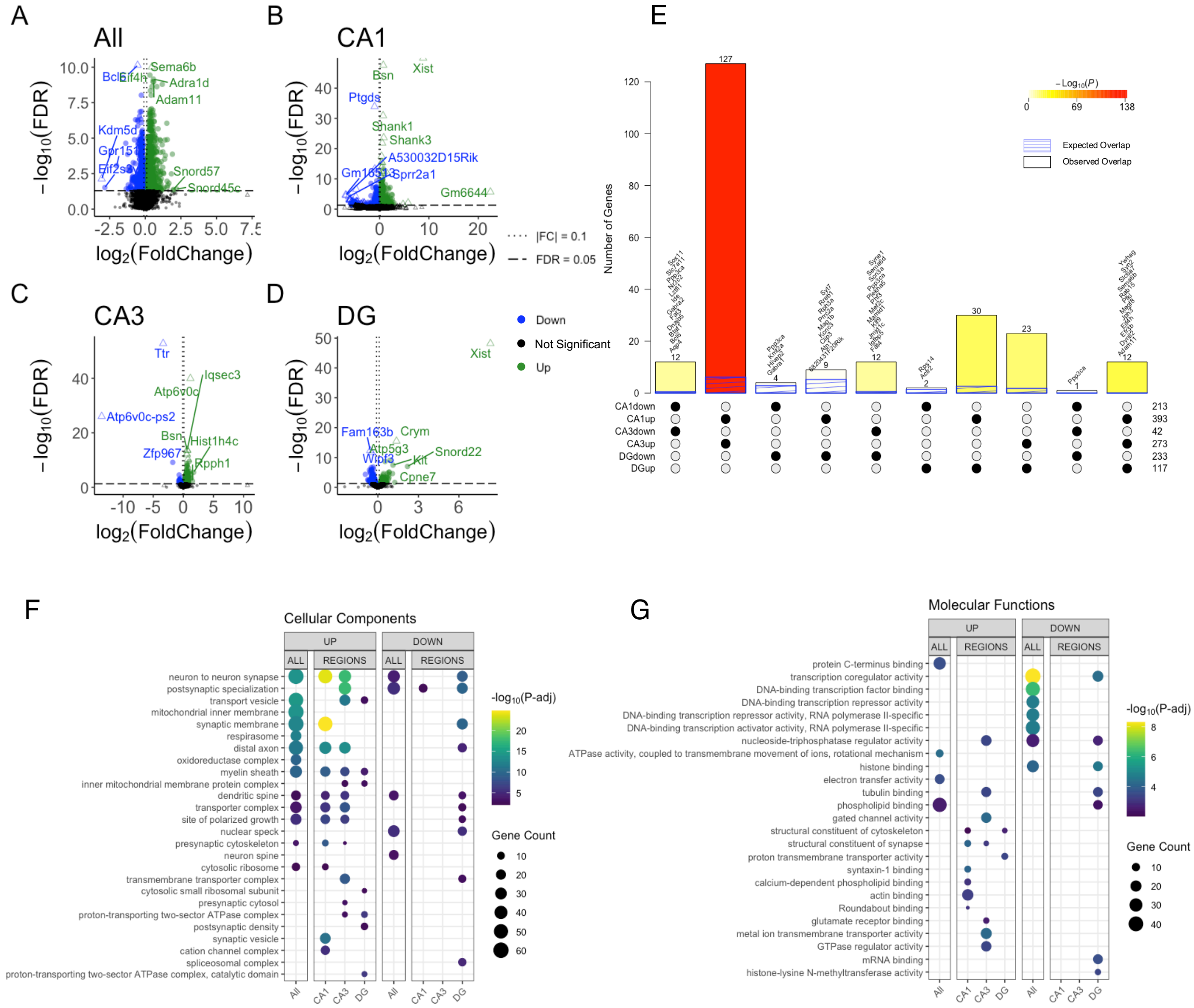

Supplemental Figure 1. Trained CA1 and CA3 subregions express similar DEGs and postsynaptic cellular component GO terms

A-D) Volcano plot of DEGs between trained and untrained CA1, CA3 and DG samples, combined and individually. Triangular points mark data out of the frame of the main text figure. E) Upset plot of overlaps in regional DEGs demonstrates greatest similarity between CA1 and CA3 samples. DEGs from each regional analysis were stratified by direction of fold change. In the upset plot, each bar represents the number of genes in an intersection of lists of DEGs. The intersecting lists is represented by the dots underneath the bar. The hashed line on each bar

bioRxiv preprint doi: https://doi.org/10.1101/2023.12.30.573225; this version posted January 1, 2024. The copyright holder for this preprint (which was not certified by peer review) is the author/funder, who has granted bioRxiv a license to display the preprint in perpetuity. It is made available under aCC-BY 4.0 International license.

represents the expected intersection of two identically sized random lists. Overlaps were tested with Fisher's exact test using the list of all expressed genes as a background. The color of the bar represents the $-\log_{10}(P)$ for the BH corrected P-value of each intersection. F,G) Cellular compartments (F) and molecular functions (G)overrepresented amongst DEGs detected between the hippocampal subregions from trained and untrained samples for all regions combined and separated CA1, CA3 and DG samples. Dot color reflects the statistical significance ($-\log_{10}(FDR)$) of the biological process enrichment. Dot size reflects the number of detected DEGs mapped to the genes involved in a given biological process.

bioRxiv preprint doi: https://doi.org/10.1101/2023.12.30.573225; this version posted January 1, 2024. The copyright holder for this preprint (which was not certified by peer review) is the author/funder, who has granted bioRxiv a license to display the preprint in perpetuity. It is made available under aCC-BY 4.0 International license.

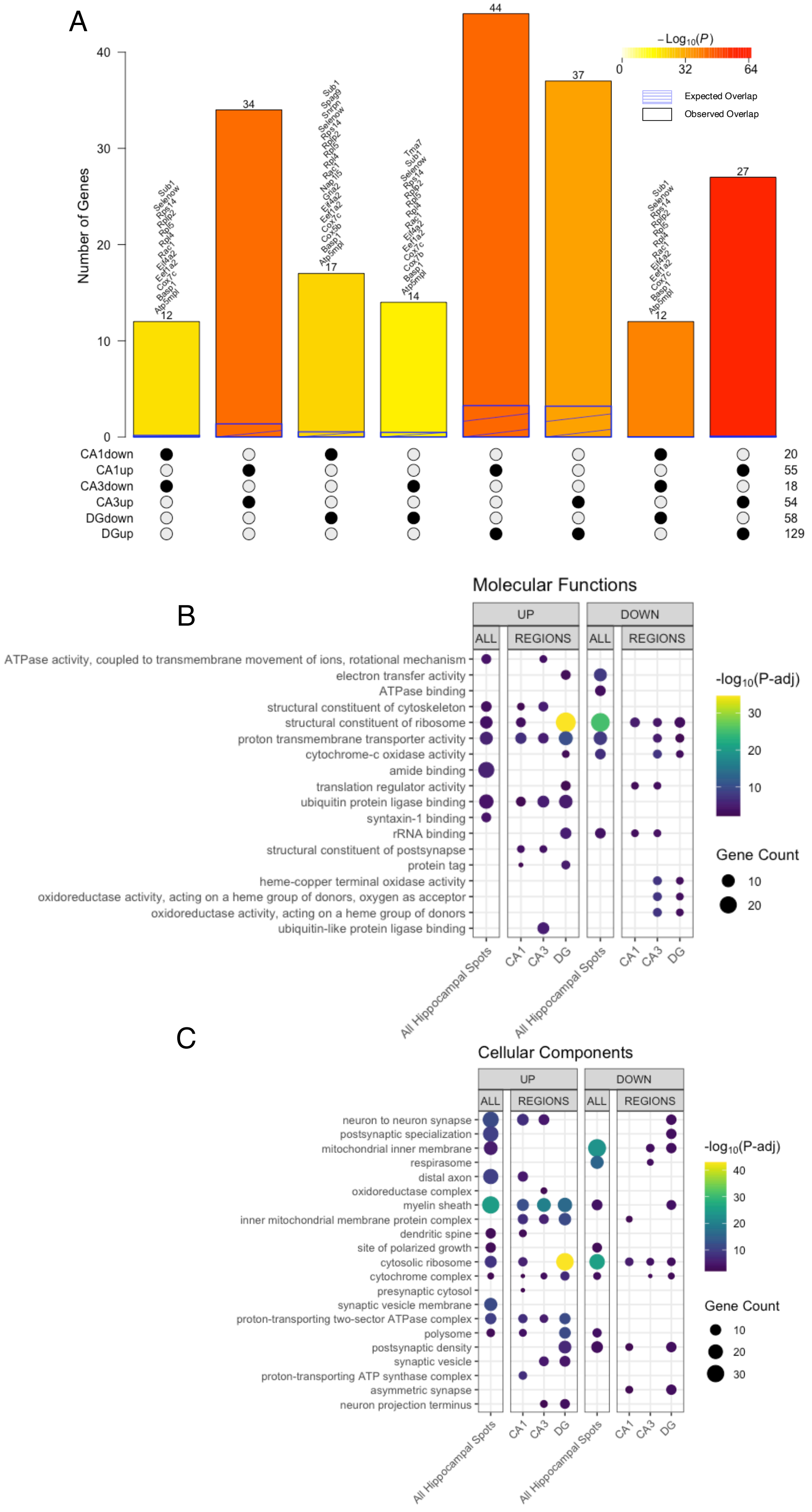

bioRxiv preprint doi: https://doi.org/10.1101/2023.12.30.573225; this version posted January 1, 2024. The copyright holder for this preprint (which was not certified by peer review) is the author/funder, who has granted bioRxiv a license to display the preprint in perpetuity. It is made available under aCC-BY 4.0 International license.

Supplemental Figure 2. Cellular components and molecular functions enriched among DEGs detected using spatial transcriptomics

A) Upset plot of overlaps in regional DEGs demonstrates greatest similarity between CA1 and CA3 subregions. DEGs from each regional analysis were stratified by direction of fold change. In the upset plot, each bar represents the number of genes in an intersection of lists of DEGs. The intersecting lists is represented by the dots underneath the bar. The hashed line on each bar represents the expected intersection of two identically sized random lists. Overlaps were tested with Fisher's exact test using the list of all expressed genes as a background. The color of the bar represents the $-\log_{10}(P)$ for the BH corrected P-value of each intersection. B,C) Molecular functions (B) and cellular compartments (C) overrepresented amongst DEGs detected between the hippocampal samples from trained and untrained samples for all hippocampal spots and spots in the cell layers of CA1, CA3 and DG subregions. Dot color reflects the statistical significance ($-\log_{10}(FDR)$) of the biological process enrichment. Dot size reflects the number of detected DEGs mapped to the genes involved in a given biological process.

bioRxiv preprint doi: https://doi.org/10.1101/2023.12.30.573225; this version posted January 1, 2024. The copyright holder for this preprint (which was not certified by peer review) is the author/funder, who has granted bioRxiv a license to display the preprint in perpetuity. It is made available under aCC-BY 4.0 International license.

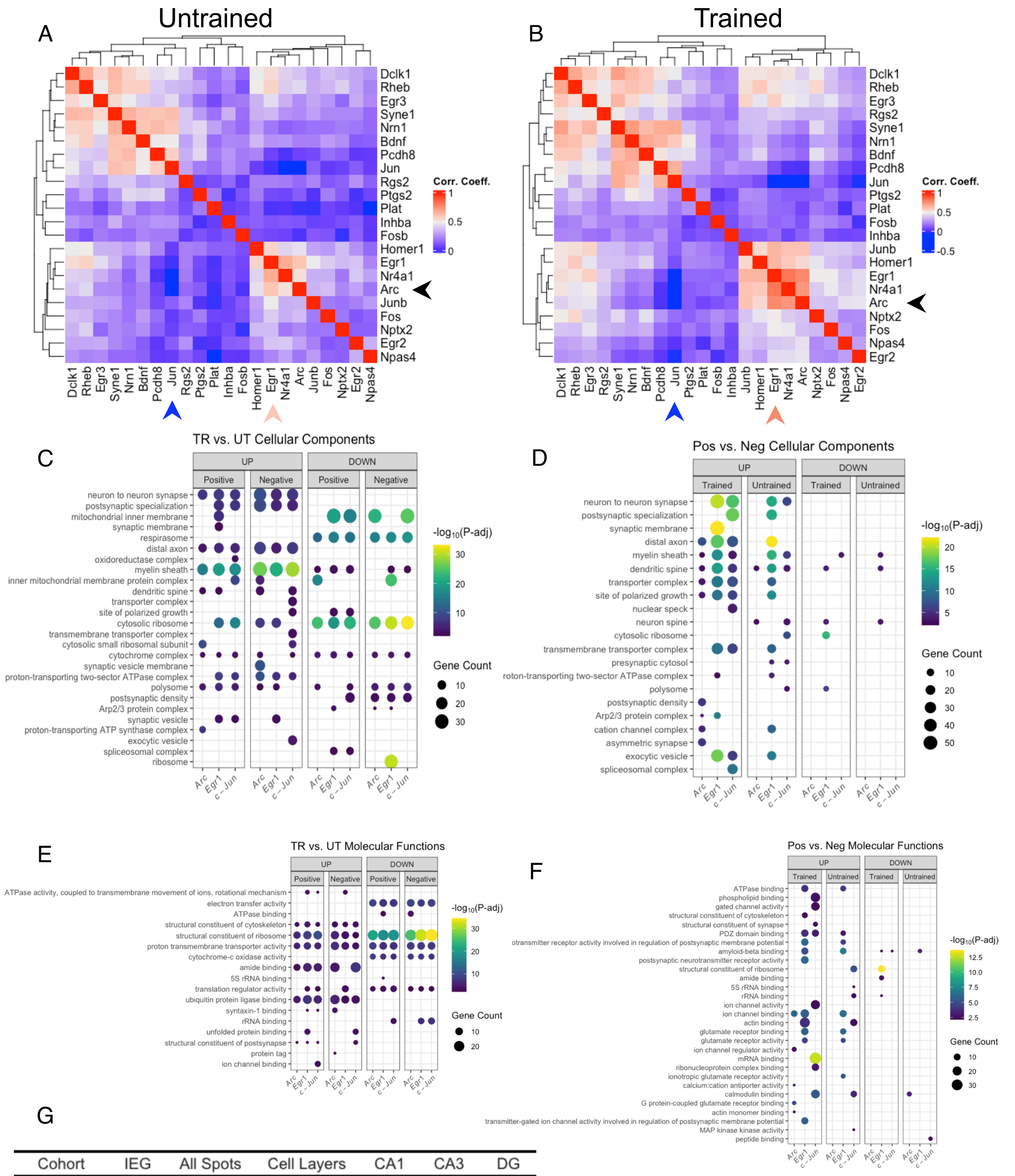

G

Cohort	IEG	All Spots	Cell Layers	CA1	CA3	DG
Trained	Arc	195/542	56/146	34/49	20/43	2/54
	Egr1	380/542	130/146	48/49	41/43	41/54
	c-Jun	308/542	106/146	22/49	32/43	52/54
Untrained	Arc	169/568	58/155	34/51	19/42	5/62
	Egr1	256/568	113/155	51/51	33/42	29/62
	c-Jun	175/568	83/155	12/51	18/42	53/62

bioRxiv preprint doi: https://doi.org/10.1101/2023.12.30.573225; this version posted January 1, 2024. The copyright holder for this preprint (which was not certified by peer review) is the author/funder, who has granted bioRxiv a license to display the preprint in perpetuity. It is made available under aCC-BY 4.0 International license.

Supplemental Figure 3. The expression of the IEGs *Egr1* and *c-Jun* are most and least correlated with the expression of *Arc*, respectively

A, B) Heatmaps reflecting the pairwise Pearson correlation coefficients between IEGs detected in the Trained (A) and Untrained (B) sample. Pearson correlation between *Arc* and *Egr1* or *c-Jun* are marked by the caret on the x-axis colored to reflect the value of the correlation coefficient. The correlation coefficients for *Arc* and *Egr1* are r(21) = .598, p = 2.24 x 10^{-56} in the untrained sample and r(21) = .764, p = 5.45 x 10^{-105} in the trained sample. The correlation coefficients for *Arc* and *c-Jun* are r(21) = -.057, p = 0.17 in the untrained sample and r(21) = -.105, p = 0.014 in the trained sample. C,E) Molecular functions (C) and cellular compartments (E) overrepresented amongst DEGs detected across trained and untrained samples for spots expressionr the IEGs *Arc*, *Egr1*, and *c-Jun*. Dot color reflects the statistical significance ($-\log_{10}$(FDR)) of the biological process enrichment. Dot size reflects the number of detected DEGs mapping to the gene belonging to a given biological process D,F) Molecular functions (D) and cellular compartments (F) overrepresented amongst DEGs detected between IEG positive and negative spots within either the trained or untrained sample. Dot color reflects the statistical significance ($-\log_{10}$(FDR)) of the biological process enrichment. Dot size reflects the number of detected DEGs mapping to the gene belonging to a given biological process G) Table total number of IEG expressing spots relative to the total number of hippocampal spots for each area.

bioRxiv preprint doi: https://doi.org/10.1101/2023.12.30.573225; this version posted January 1, 2024. The copyright holder for this preprint (which was not certified by peer review) is the author/funder, who has granted bioRxiv a license to display the preprint in perpetuity. It is made available under aCC-BY 4.0 International license.

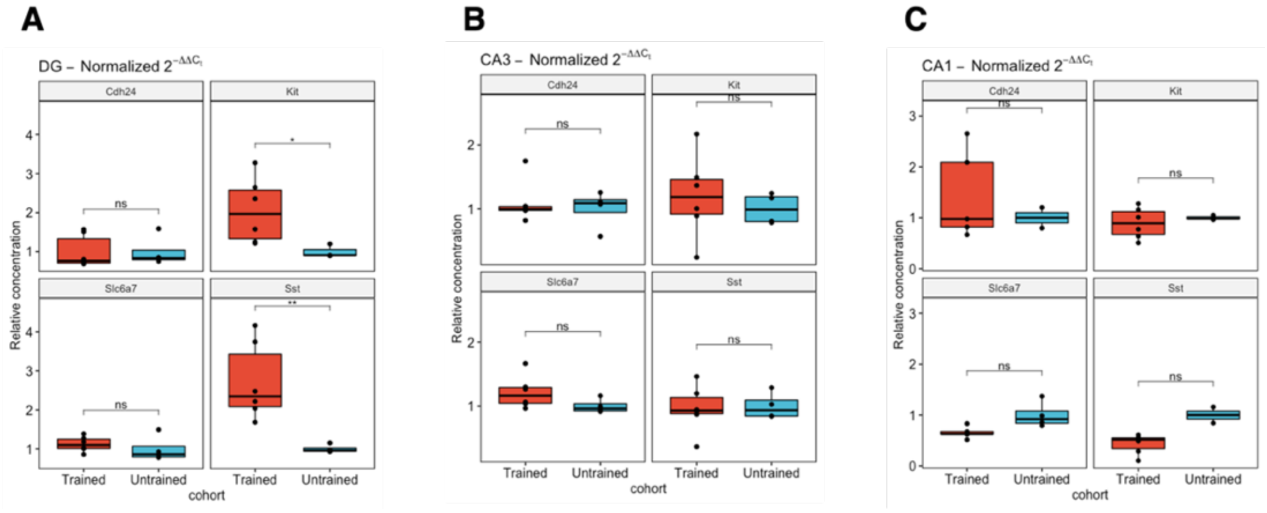

Supplemental Figure 4. Relative expression of select DEGs through RT-qPCR

A-C)Average normalized relative expression ($2^{-\Delta\Delta Ct}$) of genes in trained samples (red) as compared to untrained samples (blue) in dentate gyrus (A), CA3 (B) and CA1 (C) subregions. n= 6 trained and n=4 untrained animals. Student t-test was performed between ΔCt values from trained and untrained samples for each gene.
